# Around the Hospitals

**Published:** 1895-09-14

**Authors:** 


### Around the Hospitals.

[Notice to the Managers of Institutions.—Special space is now reserved for the insertion of notices of hospital meetings and festivals. The Editor requests that all notices may reach The Hospital Office, 428, Strand, at least one week before the date of each meeting.]

**The Royal Hospital, Richmond.**—Although the annual subscribers to the hospital remain far too few in number, and indeed continue to decrease, the past year proved a prosperous one to the institution, leaving it with a balance to the good on its accounts. This was due to large bequests, an unstable form of income. The improvements and additions are still progressing, and when completed will depend on a larger generosity from the neighbourhood for their maintenance. The report contains excellently drawn up comparative tables, which give full information. The in-patients during the past year amounted to 383, the out-patients to 3,119. The cost per in-patient was £6 11s. 11½d., a large figure even for a small hospital, and the cost per bed to £76 11s. 7d, which is also very high. The income amounted to £3,656 8s. 2d., and the expenditure to £2,945 10s.

**Prince Alfred Hospital, Sydney.**—During 1894 2,927 patients were under treatment, as against 2,611 in 1893. Of the total number admitted during the year, 787 were cases of accident or emergency, 1,043 were received under Government orders, for which the hospital receives 21s. per week towards their maintenance, and 901 contributed more or less towards their support. The mean residence of patients was 27 days. The rate of mortality was 9·74, against 9·92 in 1893. The attendance of outdoor and casualty cases was 35·136, against 31·258 in 1893. The total amount contributed by patients during the year was £2,492 6s. 3d., being £434 3s. 4d. less than in the preceding year, such diminution being thought attributable to the present industrial depression. The number of typhoid cases was 161, against 82 the previous year. The total number of operations performed was 1,262. The average cost per occupied bed was £69 1s. 3d. This average is obtained by deducting all moneys paid for alterations and repairs and the estimated cost of the out-patients' department.

**The Lancaster Infirmary.**—The new infirmary for Lancaster will be much appreciated by those now working in the old institution, where pressure on space is an ever-increasing difficulty. The new buildings are progressing; some of the structure is finished, and it is hoped that all will be completed next year. The sum of £6,500 is still needed before this can be done. The infirmary is in a fairly prosperous financial condition, but it must be borne in mind that the uncertain source, bequests, has brought this about, and not the more satisfactory annual subscriptions. The working men's collections and contributions are a more satisfactory cause of prosperity, and the infirmary must congratulate itself on the great interest taken in it by the workpeople's collection committee. The report for the past year contains a well drawn out table showing the cost per bed and how the expenses are apportioned. It is very instructive, and we commend it to hospital secretaries generally. In the table the manner of crediting expenses to out-patients is given, and on the present occasion 2s. 5d. is the sum per head arrived at; and as the cost per in-patient is not unusually low, it may be regarded as a fair sample of averaging for small hospitals. At the Essex and Colchester Hospital for instance, where patients are more numerous, over 4s. is given as the average cost of each out-patient, and the cost per in-patient is not low. During the past year 343 in-patients and 2,772 out-patients were received. The income amounted to £2,155, and the expenditure to £1,851.

